# Hepatic anisakiasis mimicking metastatic liver tumour

**DOI:** 10.1016/j.ijscr.2019.06.010

**Published:** 2019-06-12

**Authors:** Ryosuke Kita, Hiroki Hashida, Kenji Uryuhara, Satoshi Kaihara

**Affiliations:** Department of Surgery, Kobe City Medical Center General Hospital, Japan

**Keywords:** Anisakis, Hepatic anisakiasis, Extragastrointestinal anisakiasis, Metastatic liver cancer, Rectal cancer

## Abstract

**Introduction:**

Anisakiasis is a parasitic disease caused by anisakid nematode larvae, which are found in raw or undercooked fish. It occurs more frequently in the digestive tract, but has also been reported outside the gastrointestinal tract. This report details a case of rare hepatic anisakiasis that was initially diagnosed as metastatic rectal cancer.

**Presentation of case:**

A 60-year-old male underwent radical resection for stage III rectal cancer followed by adjuvant chemotherapy for six months. Seven months after surgery, a new, 10 mm diameter area of low density was seen in segment 4/8 margin of the liver on enhanced computed tomography (CT) scan. Metastatic liver tumour from rectal cancer was suspected. Laparoscopic partial hepatectomy was performed. The diagnosis was revised to hepatic anisakiasis when pathological examination revealed anisakis simplex larvae in the necrotic tissue.

**Discussion:**

Anisakiasis outside of the gastrointestinal tract is a rare finding, but may be seen more frequently with the recent increase in the consumption of raw fish around the world. Hepatic anisakiasis may mimic tumours in the liver. Clinicians should consider dietary inquiry and further evaluation in patients with liver margin tumours less than 20 mm in size that are not positively malignant, as hepatic anisakiasis may be a possible diagnosis.

**Conclusion:**

We report a rare case of hepatic anisakiasis which initially presented as suspected metastatic cancer. Hepatic anisakiasis should be considered in the differential diagnosis of low density liver tumours less than 20 mm, where the patient’s diet includes raw fish.

## Highlights

• Anisakiasis is a parasitic disease caused by anisakid nematode larvae in raw fish.
• Anisakiasis may increase with growing consumption of raw fish around the world.
• Hepatic anisakiasis presents as small, low density tumours in the liver margin.
• The lesions resemble recurrent carcinoma, and can lead to unnecessary surgery.
• Dietary investigation is warranted when liver tumour malignancy is not certain.

## 1 Introduction

Anisakiasis is the zoonotic disease caused by the third larval stage of anisakid nematodes, most frequently belonging to the Anisakis, Pseudoterranova genera and species of the genus Contracaecum [1]. This zoonotic infection is acquired through the consumption of raw or undercooked squid, mackerel and cod [2]. This is a relatively common diagnosis in East Asia due to the predominant food culture. However, with the increasing popularity of Japanese cuisine, the incidence of anisakiasis has become more frequent around the world due to the increased consumption of raw fish and mackerel [[3], [4], [5]]. More than 99% of anisakiasis cases occur in the gastrointestinal tract, specifically in the stomach and small intestine, however the larvae can penetrate the gastrointestinal mucosa or other organs with clinical consequences and potentially allergic reactions [6].

There are two types of allergic reaction, including relaxation type with primary infection and fulminant type with reinfection. At the time of primary infection, the allergic reaction is weak and there are few symptoms. In extragastrointestinal anisakiasis, anisakis larvae from the primary infection penetrate the wall of the gastrointestinal tract asymptomatically, and sometimes granulomatous tissues are formed in the intraperitoneal organs including the omentum, mesentery, peritoneum, abdominal wall, lymph nodes, liver, pancreas, and ovaries.

Hepatic anisakiasis is said to be about 0.2% of all cases [[7], [8], [9]]. To our knowledge, there have been no reports of hepatic anisakiasis outside of Japan, and there is not yet a clear description of the imaging characteristics of the disease. Since the possibility that hepatic anisakiasis will occur in other countries is high, it is important to describe any diagnostic factors. We provide details of our case of hepatic anisakiasis which developed as a tumour at the border of the liver, and summarize the presentations of previously reported cases.

The work has been reported in line with the SCARE criteria [10].

## 2 Presentation of case

**Fig. 1:**
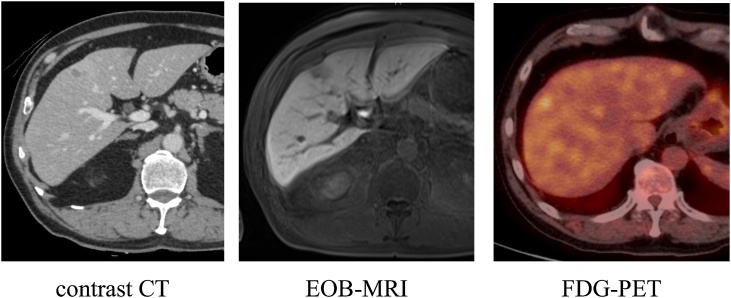
Images of the liver. A: A 10 mm area of low density is observed on the surface in segment 8 of the liver in contrast CT. B: EOB-MRI shows a decrease in uptake of EOB. C: Abnormal FDG accumulation of SUV max 2.8 is seen on PET examination. CT: computer tomography. EOB MRI: gadoxetic acid magnetic resonance imaging. FDG: fluoro-deoxyglucose. SUV: standardized uptake value. PET: positron emission tomography.

**Fig. 2:**
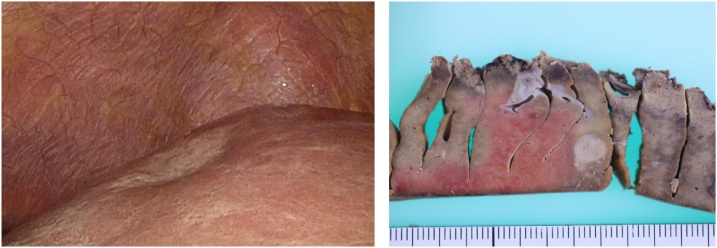
Intraoperative findings and resected specimen. A: Intraoperative findings show a white fleck in segment 8 of the liver. B: An 8 mm nodule of white necrosisis found in the resected specimen right under the serous membrane.

**Fig. 3:**
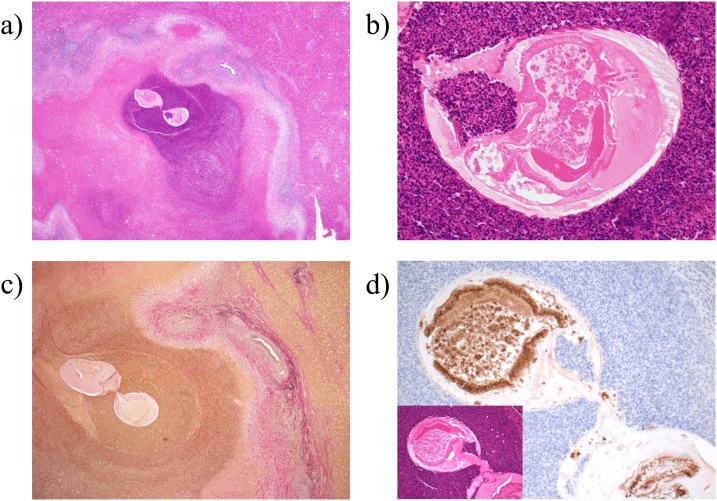
A: A nodule with surrounding necrosis and inflammatory cell infiltration is observed in the vicinity of the serosa. B: Structures considered to be worm bodies are seen in the nodule. C: The worm body is shown intravenously by EVG staining. D: The worm body shows positive immunostaining against anisakis antigen. EVG: elastic-van Giesen.

The patient was a 60-year-old man who had been diagnosed with rectal cancer. He underwent laparoscopic radical low anterior rectal resection and construction of a covering ileostomy. Pathological examination revealed that the rectal cancer was pT3 (A), pN1b (2/17), cM0, pStage IIIB. To prevent recurrence, he received postoperative adjuvant chemotherapy of modified FOLFOX (fluorouracil, l-leucovorin and oxaliplatin) for 6 months after surgery. Follow-up computed tomography (CT) performed at 7 months after surgery detected a new low density area of 10 mm in diameter in the surface of liver segment 4/8, and a deformation in which the liver surface in the vicinity was recessed. The patient showed no symptoms such as abdominal pain during follow-up period after surgery. Gadoxetic acid magnetic resonance imaging (EOB-MRI) revealed nodules of about 8 mm on the surface of liver segment 4/8 and a surrounding area of abnormal EOB uptake. Positron emission tomography (PET)-CT was performed to confirm the presence of a malignant liver tumour and to search for other metastasis. The liver lesion had localized light fluorodeoxyglucose (FDG) uptake in the delayed phase (standardized uptake value (SUV) max: 2.8) and other distant metastasis was not detected (Fig. 1). Serum tumour markers including CEA and CA19-9 were not elevated, and were similar to the levels before initial chemotherapy. These findings were compatible with a metastatic tumour from rectal cancer. Because the mass was judged to be resectable, laparoscopic partial hepatectomy and ileostomy closure was planned with a diagnosis of liver metastasis from rectal cancer. Intraoperative findings revealed a slight white, crude tumour on the liver segment 8 surface with no other lesions recognized, so partial hepatectomy of segment 8 was performed. The patient recovered postoperatively and was discharged on postoperative day 7. A nodule of approximately 8 mm in diameter was macroscopically identified in the resected specimen (Fig. 2). Necrotic granulation tissue of approximately 5 mm was found in the centre of the nodule, accompanied by a few polygonal giant cells at the margin on the microscopic examination. A parasitic worm body was recognized with Elastica van Gieson staining in a structure suspected to be a vein. Grocott staining did not reveal any fungus or protozoon infection, and the worm body was finally proved to be anisakis simplex by anisakis antibody staining (Fig. 3). It was thought that the anisakis larvae broke through the intestinal wall and invaded the vein from the liver surface. Detailed inquiry after surgery revealed that the patient had a history of eating raw fish. Thereafter, no new lesions have appeared in the liver and there has been no recurrence of rectal cancer.

## 3 Discussion

Two important facts were derived from this case: firstly the existence of hepatic anisakiasis, and secondly, the possibility of hepatic anisakiasis should be considered with low density liver margin tumours of less than 20 mm by contrast CT if the patient has a history of eating raw fish.

The first zoonotic case attributed to the anisakis species was described in the Netherlands by Van Thiel et al. in 1960, and anisakiasis is divided into three types; gastric, intestinal, and extragastrointestinal anisakiasis [11]. Ishikura et al reported that gastric anisakiasis accounts for 95.7% of all cases, intestinal anisakiasis 3.8%, and extragastrointestinal anisakiasis just 0.5% of all cases. Among extragastrointestinal anisakiasis, hepatic anisakiasis is extremely rare. Only 12 cases of hepatic anisakiasis have been reported previously (Table 1). To the best of our knowledge, our case was the 13th case in the world. As in this case, most of extra-gastrointestinal anisakiasis including hepatic anisakiasis are asymptomatic and detected incidentally by imaging studies. In primary infection, anisakis larvae penetrate the wall of the gastrointestinal tract with no symptoms and form abscesses and granulomas in intraperitoneal organs [8].

**Table 1:** Reported cases of hepatic anisakiasis.

Author	Age (Years)	Sex	Localization	Size (mm)	CT findings
Ueki	62	M	Right lobe surface	2	None
Kagei	51	M	unknown	15	None
Kawakami	58	M	S8 surface	20	LDA
Kunitomi	34	F	S4 surface	20	LDA
Ideguchi	68	F	S4/5	14	LDA
Morita	59	F	S2 surface	20	LDA
Ishida	64	M	S8 surface	20	LDA
Hayashi	70	M	S8 surface	8	LDA
Irie	unknown	unknown	unknown	22	LDA
Nogami	44	F	S4 surface	15	LDA
Sekoguchi	63	M	S6 surface	4	None
Murata	28	M	S7 surface	12	LDA
Our case	60	M	S8 surface	8	LDA

CT: computed tomography. LDA: low density area.

Hepatic anisakiasis may occur as anisakis larvae migrating from the digestive tract wall into the peritoneal cavity invade the liver surface and form a tumour at the liver margin. It is also speculated that larvae from the gastrointestinal tract might reach the liver via the portal vein, but there are no reports of hepatic anisakiasis invasion routes from any other path than the liver surface.

In our case, anisakis larvae were recognized in the hepatic vein. Although pathological findings could not show clear evidence of invasion from the liver surface, we judged that anisakis larvae penetrated the liver surface from intraoperative findings which confirmed liver surface contractions at the tumour site and pathological findings in which necrotic tissue and granulation tissue spread around the liver surface.

In general, hepatic anisakiasis is difficult to make clinical diagnosis simply by using imaging modalities. In all presented cases including our case, precise diagnosis based on imaging studies was not achieved before pathological examination. Even after pathological examination, to make a definitive diagnosis is also difficult because larva body has often collapsed. The infection in human body is transient and the parasite will die within 14 days, leaving a persistent inflammation forming granuloma. Various immunologic assays like complement fixation test, immunofluorescent-antibody test, immunodiffusion test, immunoelectrophoretic assays, enzyme-linked immunosorbent assay, and radio-allergosorbent test is reliable for obtaining final diagnosis even when the larvae body collapses with time. Use of monoclonal antibodies specific to anisakis antigens also help the definite diagnosis.

Hepatic anisakiasis tumours present as a small low density area of the liver margin on CT. In previously reported cases where there was a description of localization, all tumours were at the liver margin, with an average size of 13.8 mm, and almost all tumour less than 20 mm. Low density areas in contrast CT are thought to reflect granulomas and necrotic tissue. Incidental detection of this lesion is difficult to distinguish from carcinoma recurrence in patients with a history of malignancy, and can lead to unnecessary surgery, chemotherapy, and radiotherapy. Follow-up observation or biopsy can be options for a small low density tumour at the liver margin, unless we positively suspect malignant disease.

In some cases the collapsing worm body may make the final diagnosis difficult, even after resection. Therefore, the exact frequency of hepatic anisakiasis may be higher than suspected. In our case, the dietary history of the patient and the pathological findings, including significant infiltration of inflammatory cells, oedema, formation of granulation tissue, and cuticular structures characteristic of nematodes such as anisakis and gnathostoma, were consistent with a diagnosis of anisakiasis [12].

## 4 Conclusion

Changing food cultures, including consumption of raw fish, will likely lead to an increase in anisakiasis cases in the Western countries. Hepatic anisakiasis is rare but should be a consideration if a low density small tumour of the liver margin is detected on contrast CT. The dietary history for the patient should be thoroughly evaluated. When clinical findings do not support positive suspicion of malignant tumours, close observation or biopsy may prevent excessive surgical invasion.

## Declaration of Competing Interest

The authors have no conflict of interest.

## Sources of funding

This study has not received any funding.

## Ethical approval

This case report was approved by the committee of our institute.

## Consent

Written informed consent was obtained from the patient.

## Author contribution

Ryosuke Kita – Author, Editing of manuscript.

Hiroki Hashida – contributor.

Kenji Uryuhara – contributor.

Satoshi Kaihara – contributor.

## Registration of research studies

None.

## Guarantor

The Guarantor is Ryosuke Kita.

## Provenance and peer review

Not commissioned, externally peer-reviewed.
